# Pre- to postoperative coagulation profile of 307 patients undergoing oesophageal resection with epidural blockade over a 10-year period in a single hospital: implications for the risk of spinal haematoma

**DOI:** 10.1186/s13741-017-0070-7

**Published:** 2017-10-04

**Authors:** Owain Thomas, Emanuel Lybeck, Per Flisberg, Ulf Schött

**Affiliations:** 1 Department of Anaesthesiology and Intensive Care Medicine, Institute of Clinical Sciences, Lund, Medical Faculty, University of Lund, 221 00 Lund, Sweden; 2 Department of Paediatric Anaesthesia and Intensive Care, SUS Lund University Hospital, 22185 Lund, Sweden; 3 Oskarshamn Hospital, 572 28 Oskarshamn, Sweden; 4 Department of Anaesthesia and Intensive Care, Helsingborg Hospital, Södra Vallgatan 5, 254 37 Helsingborg, Sweden; 5 Department of Anaesthesia and Intensive Care, SUS Lund University Hospital, 221 85 Lund, Sweden

**Keywords:** Epidural anaesthesia, Routine coagulation testing, Spinal haematoma

## Abstract

**Background:**

Epidural anaesthesia and analgesia are indicated for oesophageal surgery. A rare but serious complication is spinal haematoma, which can occur on insertion, manipulation or withdrawal of catheters. Evidence and guidelines are vague regarding which tests are appropriate and how to interpret their results. We aimed to describe how routine coagulation test results change during oesophagectomy’s perioperative course.

**Methods:**

Following ethical approval, we retrospectively identified patients who had undergone oesophagectomy between 2002 and 2012. Blood test results and details of operations, haemorrhage and complications were recorded and analysed with Excel and R. A literature search was conducted using the PubMed terms ‘epidural’ AND ‘coagulation’ AND English language. Relevant articles published in 2000 and after were included.

**Results:**

Three hundred and seven patients received a thoracic epidural infusion with bupivacaine and morphine while 51 received an intravenous morphine infusion. Tests taken preoperatively and before the planned withdrawal of the epidural catheter demonstrated increases in all three measures: aPTT (activated partial thromboplastin time), PT-INR (prothrombin international normalised ratio) and platelet count (Plc). Postoperative thrombocytopenia was almost non-existent while aPTT or PT-INR was elevated above the reference range in 129/307 patients: aPTT was elevated in 116/307 while PT-INR was elevated in 32/307. This is too small a sample to allow meaningful estimation of risk of spinal haematoma: it may be as high as 2.3%. The literature search returned 275 articles, of which 57 were relevant. Twenty-one concerned the natural history of postoperative coagulation; 16, the incidence of and risk factors for spinal haematoma; and 5, evaluation of specific blood tests. Postoperative coagulation is characterised by thrombocytosis and transient moderately abnormal routine coagulation test results. Viscoelastic tests are not validated in the stable postoperative setting.

**Conclusions:**

Screening for coagulopathy before removal of epidural catheters is of unclear benefit since elevated aPTT and PT-INR are usual and may not indicate hypocoagulation. A thorough clinical assessment is important. We nevertheless recommend caution when being presented with elevated routine tests of coagulation before withdrawing an epidural catheter: viscoelastic haemostatic tests may have a role in testing before withdrawal of epidural catheters but they are so far not validated. Future research should include advanced coagulation analysis as soon as a patient is unfortunate enough to have a spinal haematoma.

**Electronic supplementary material:**

The online version of this article (10.1186/s13741-017-0070-7) contains supplementary material, which is available to authorized users.

## Background

Epidural anaesthesia and analgesia may reduce the risk of morbidity and mortality in patients undergoing oesophageal surgery, which can be a major insult for patients who are often already frail (Merritt et al. 2011; Watson and Allen 1994). An uncommon but serious complication of epidural catheterization is spinal haematoma, which most often occurs at the time of catheterization or withdrawal of catheters (Moen et al. 2004). For this reason, guidelines generally recommend withdrawal of catheters when routine tests of coagulation, prothrombin time international normalised ratio (PT-INR), activated partial thromboplastin time (aPTT) and platelet count (Plc), are within their normal ranges (Breivik et al. 2010; Horlocker et al. 2010; Gogarten et al. 2010).

The problem with these recommendations is that it is unclear what to do when results indicate mild hypocoagulation: should treatments such as vitamin K, plasma transfusion or prothrombin complex concentrate be given to normalise a PT-INR of 1.5? Should thrombosis prophylaxis be withheld or the catheter be left in place because of a slightly prolonged aPTT, potentially exposing the patient to the risk of venous thrombosis or catheter infection? And should a platelet transfusion and its side-effects be given to correct a Plc of 99 × 10^6^/ml before withdrawal of an epidural catheter in a patient otherwise recovering well?

Most published articles concerning coagulation and postoperative epidural analgesia after major surgery concern hepatic resection where liver function may be affected. In this study, we present pre- and postoperative coagulation status from patients who underwent oesophageal resection over a period of 10 years. Our hypotheses were that postoperative coagulation tests would indicate hypocoagulation, and that postoperative serum creatinine would be correlated to elevated aPTT suggesting an accumulation of thrombosis prophylaxis.

We also present a review of the literature aiming to establish whether our results agreed with previous research, whether there is evidence that current coagulation tests can be used to estimate the risk of spinal haematoma and why coagulation parameters may be elevated in the postoperative period.

## Methods

We included patients who had between 2002 and 2012 received epidural or intravenous analgesia after oesophageal surgery at our tertiary hospital, which is a regional centre for treatment of oesophageal cancer. Patients were identified using filed prescription sheets from epidural or intravenous infusion pumps. The data shown in Table 1 was recorded for each patient, which included routine coagulation status before surgery, and before withdrawal of epidural catheters.

**Table 1:** Summary of data collected for each patient

Patient details	Preoperative data	Data prior to withdrawal of epidural catheter
Age	Time of epidural catheterization	Time of withdrawal of epidural catheter
Sex	Time of blood testing	Time of blood testing
Epidural or intravenous analgesia	Activated partial thromboplastin time (aPTT)	Time and dosage of the last dose of low molecular weight heparin (LMWH) before withdrawal of epidural catheter
Reason for intravenous analgesia if applicable	Prothrombin time international normalised ratio (PT-INR)	Activated partial thromboplastin time (aPTT)
Level of epidural catheterization	Platelet count (Plc)	Prothrombin time international normalised ratio (PT-INR)
	Blood haemoglobin level (Hb)	Platelet count (Plc)
	Serum albumin (Alb)	Blood haemoglobin level (Hb)
	Serum creatinine (Crea)	

Patients were attributed an identification number and masked data was entered in to a spreadsheet using Microsoft Excel before export to R for statistical analysis (R version 3.3.1, www.r-project.org). Original data is available in Additional file 1 but we have removed patients’ ages and the date of operation to preserve anonymity. The significance of differences between pre- and postoperative test results were tested using the paired Student’s *t* test.

**Fig. 1:**
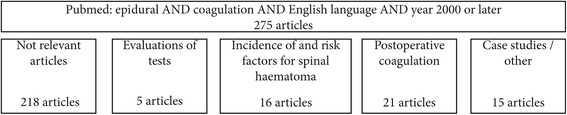
Search strategy for the literature review presented in this article

Figure 1 summarises the literature search. A PubMed search was carried out using the terms “epidural” AND “coagulation” AND English language. Articles concerning coagulation in the context of perioperative epidural analgesia published in 2000 or later were retrieved. Articles were sorted into the topics shown in Fig. 1.

## Results

Three hundred and seven patients, 69 women and 238 men, who received a thoracic epidural catheter immediately before induction of general anaesthesia for oesophageal surgery were included. Additional file 1 displays patient and quality data: the median length of time between insertion and withdrawal of catheters was 8.5 days (range 1–38). The mean level of placement was T8 (range T4–T12). The median intraoperative haemorrhage was 500 ml (range 50–3800 ml). Forty-one of the patients who had received an epidural catheter were postoperatively given a patient-controlled intravenous morphine infusion, for reasons which are shown in Table 2. This represents 13% of the patients enrolled. All patients were treated with thrombosis prophylaxis (enoxaparin 40 mg daily other than four patients who received 80 mg). No serious complication of epidural catheterization was recorded.

**Table 2:** Reasons for replacing epidural analgesia with intravenous analgesia with an intravenous patient-controlled morphine infusion

Inadequate analgesia provided by epidural catheter	23
Unclear from patient notes	7
Accidental withdrawal of the catheter	7
Elective transition from epidural to IV-PCA	6
Leakage around the catheter	2
Suspected infection around the catheter	2
‘Sudden chest pain’, epidural catheter presumably withdrawn before antiplatelet drugs	1

An additional 51 patients who underwent oesophageal resection were identified, who did not receive an epidural catheter. Six proved difficult to catheterize such that the attempt to insert an epidural catheter was given up. Nine of the 51 did not receive an epidural because of abnormal preoperative routine coagulation test results while one had known von Willebrand’s Disease, one scleroderma and one renal failure. Ten had relative anatomical contraindications.

**Fig. 2:**
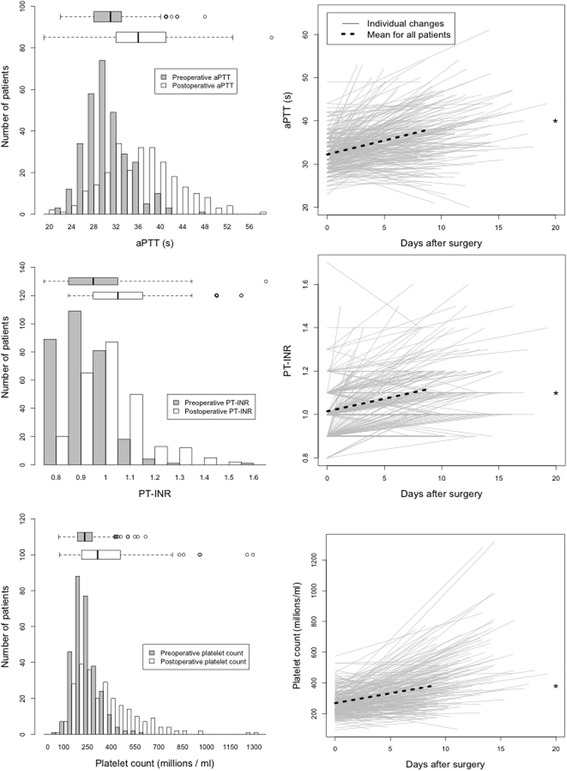
Dynamics of routine coagulation status between insertion and withdrawal of epidural catheters. Each solid grey line represents a patient. The beginning of the line at time = 0 represents results taken preoperatively, while each line ends at the time at which the patients’ epidural catheter was taken. Note that only two data points are shown for each patient. The dotted black lines show mean pre- to postoperative changes for the whole patient group and stars indicate a significant pre- to postoperative difference using the paired Student’s *t* test (*P* < 0.05). aPTT, activated partial prothrombin time. PT-INR, prothrombin time international normalised ratio

Figure 2 shows the dynamics of routine coagulation test results between insertion and withdrawal of catheters. Mean PT-INR increased significantly from 1.0 ± 0.11 to 1.1 ± 0.14: mean aPTT increased significantly from 32 ± 4.0 to 38 ± 6.7 s and mean Plc increased significantly from 269 ± 83 × 10^6^ to 339 ± 196 × 10^6^. 32/307 patients’ PT-INR was elevated above the reference range at the time of catheter withdrawal; for aPTT, this figure was 129/307; and there was only one patient whose Plc was below the reference range at this time. There was no relationship between the amount of intraoperative haemorrhage and any of the postoperative coagulation parameters.

Applying Hanley’s simple formula to the 129 patients whose aPTT or PT-INR was elevated above the reference range, the upper 95% confidence interval for the true risk of spinal haematoma in these patients is estimated to be 3/129 = 2.3% (Hanley and Lippman-Hand 1983).

Results for preoperative serum albumin, and serum creatinine and blood haemoglobin preoperatively and at the time of withdrawal, are shown in Additional file 1. Mean serum creatinine decreased significantly from 78 ± 38 to 67 ± 31 μmol/l. There was no correlation between postoperative aPTT and serum creatinine.

**Fig. 3:**
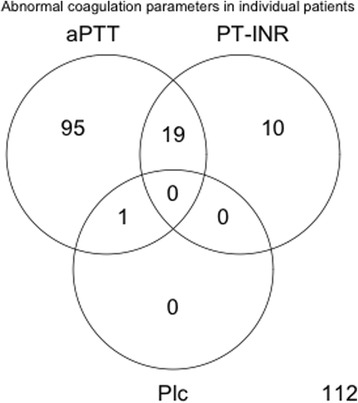
Venn diagram showing the frequency of routine coagulation tests outside their reference range at the time of withdrawing epidural catheters, indicating hypocoagulation. Only including the 237 patients for whom complete data are available: activated partial thromboplastin time (aPTT), prothrombin time international normalised ratio (PT-INR) and platelet count (Plc)

Figure 3 shows which coagulation parameters indicate hypocoagulation in the 237 patients for whom data was complete. Half of the patients had an aPTT above the upper limit of the reference range while only 10% had a PT-INR above the reference range. Thrombocytopenia was almost non-existent at the time of withdrawal of epidural catheters.

### Results of the literature search

Summaries of the relevant articles described in this section are available in Additional file 2.

#### Articles concerning the evaluation of tests in the specific context of epidural analgesia

Five articles were found. All are relatively small (between 10 and 52 subjects). The authors of this study are responsible for two of these articles (Thomas et al. 2013; Thomas et al. 2015). Four of the five articles compare whole-blood assays of coagulation to routine test results. Two of the articles find that viscoelastic tests (ROTEM®, TEG® and free-oscillation rheometry) can likely be used to monitor the effect of LMWHs on initiation of coagulation, although they cannot be considered to be validated (Thomas et al. 2015; Klein et al. 2000). Two of the articles compare PT-INR to ROTEM® or TEG® and conclude that although PT-INR is prolonged in the postoperative period after major gastrointestinal and orthopaedic surgery with or without warfarin as thrombosis prophylaxis, the viscoelastic tests indicate normo- or hypercoagulation (Thomas et al. 2013; Hepner et al. 2002). Finally, one study compared a patient-near method of measuring PT with routine laboratory results and found good concordance between the methods such that patient-near PT-INR measurement in the operating theatre or intensive care unit is realistic (Nishiyama et al. 2011).

#### Articles concerning the incidence of and risk factors for spinal haemorrhage

Sixteen articles were found. Many are prospective or retrospective studies in which routine care is given. No or few spinal haematomas are found in the population being studied, and 95% confidence limits for the true risk of haematoma are calculated. For example, among over 11,000 patients who were included, Gulur et al. identified 351 patients with abnormal routine coagulation test results when their epidural catheters were removed: two developed spinal haematomas such that the 95% confidence interval for the actual risk of spinal haematoma can be calculated to be between 1:87 and 1:2597. This statistic is complicated by the two patients developing spinal haematoma having also had spinal drains, one of which was placed because of spinal ischaemia, and that it was ‘technically difficult’, and therefore presumably traumatic, to place the catheter (Gulur et al. 2015; Ho et al. 2000). Other studies reporting series of ‘non-events’ are shown in Additional file 2: Davis et al. (2012), Franchi et al. (2011), Pumberger et al. (2013), Volk et al. (2012) and Pace et al. (2014). The results of articles lie within the interval identified from insurance claims in Sweden by Moen in 2004: the risk of spinal haematoma after epidural analgesia for orthopaedics in elderly women may be as high as 1:3600 whereas the risk in obstetrics is nearer 1:200,000. Franchi concludes that the frequency of abnormal PT-INR and aPTT in pregnant women presenting for elective neuraxial blockade is so low that these tests should not be taken (Franchi et al. 2011). Pace reaches a similar conclusion for aPTT after various operations, concluding that extremely few patients without risk factors for coagulopathy have a prolonged aPTT (Pace et al. 2014).

There are two articles which use laboratory studies to provide relevant knowledge: Kassis et al. (2000) measured aPTT and anti-FXa and anti-FIIa activity before subcutaneous heparin was given, then repeated measurements 2 and 4 h later to conclude that epidural catheters can safely be withdrawn 2 h after a subcutaneous injection of unfractionated heparin. Leonard conducted an in vitro experiment concluding that the systemic antiplatelet effect of levobupivacaine given epidurally ought not to affect coagulation, but that it may affect coagulation locally, for example in a blood patch (Leonard et al. 2000).

Two articles identify risk factors for spinal haematoma by using large retrospective studies to find and analyse relatively small numbers of patients who have had spinal haematomas. Risk factors for spinal haematoma at the time of epidural withdrawal, other than abnormal laboratory test results, include liver failure or undergoing hepatic resection, major intraoperative haemorrhage or transfusion of blood products (Pace et al. 2014), taking drugs that may affect haemostasis, such as aspirin, NSAIDs, tricyclic antidepressants or antiplatelet drugs, having abnormal anatomy, in particular spinal stenosis (Pumberger et al. 2013) but also female sex, impaired renal function and type of surgery (Volk et al. 2012).

The last category of study that sheds light on the risk and incidence of spinal haematoma is the studies which investigate epidural catheterization in categories of patient in which there is a relative contraindication for neuraxial blockade: Pastor et al. (2003) and his colleague Canto et al. (2002) describe epidural catheterization 60 min before full heparinization for extracorporteal circulation in heart surgery: over 1000 patient cases are reported without complication, giving an upper 95% confidence interval for risk of epidural haematoma of around 1:300, see Additional file 2. Similarly, Singh reports a case series of patients with FXI deficiency who received uncomplicated epidural analgesia. The most controversial study found in this review is by Liu et al. (2011), who withdrew epidural catheters from patients who had just started warfarin thrombosis prophylaxis despite the PT-INR values of up to 7. The rationale for doing this was that Benzon et al. (2010) reasons that the most important factor antagonised by warfarin is FVII and that this antagonization lags behind the PT-INR becoming prolonged. The clinical appropriateness of this reasoning is strongly questioned by Horlocker et al. (2010). Although Liu included 4365 patients, the small number of patients with PT-INR over 2 means that the upper 95% confidence limit for the actual risk of spinal haematoma can be as high as 1:250 if epidural catheters are withdrawn when PT-INR is > 2 and 1:33 when the PT-INR is > 3 (Carvalho et al. 2011).

#### Articles concerning the clinical pathophysiology of postoperative coagulation in the context of epidural analgesia

Nineteen articles were found. Fourteen studied postoperative coagulation in patients who had undergone hepatectomy. One of these also included other categories of patients. Almost all the studies recorded PT or PT-INR, aPTT and Plc daily for the first week after surgery. Almost without exception they found a transient dip in Plc during the first two to three postoperative days, accompanied by a slightly shorter prolongation of the PT and sometimes aPTT.

Tsui et al. (2004) investigated the proportion of patients with coagulopathy necessitating delaying withdrawal of an epidural catheter. The risk was highest after hepatic resection, lower in major abdominal surgery and lowest in orthopaedics. This is consistent with the other studies in this review. Risk factors for derangements in tests of coagulation were the size of liver resection, duration of surgery, preoperative liver failure, amount of intraoperative haemorrhage, requirement for blood product transfusion and low body mass index (Borromeo et al. 2000; Choi et al. 2007; Elterman and Xiong 2015; Jolly et al. 2011; Karna et al. 2015; Matot et al. 2002; Ramspoth et al. 2014; Schumann et al. 2004; Siniscalchi et al. 2004; Stamenkovic et al. 2011; Yuan et al. 2012).

Bergman and Young (2007) reply to Weinberg et al. (2006) that ‘prothrombin is not the whole story’ in postoperative coagulation after hepatic resection and point out that these patients are prone to thrombosis despite an elevated PT-INR and aPTT. This may be due to deficiencies in antithrombin or protein C, which are not detected by the PT assay. Mohammed et al. (2013) describe a prospective study of patients undergoing hepatectomy for liver donation, in which ROTEM® is compared with routine tests of coagulation. The transient hypocoagulability indicated by routine tests was not demonstrated by ROTEM® but ROTEM® did not indicate postoperative hypercoagulability either.

#### Articles presenting case reports or other relevant research

Nine relevant case studies, four reviews and a report from a postal survey were found, see Additional file 2. Two reports describe complications arising as a result of treating a laboratory test indicative of hypocoagulability, in an attempt to reduce the risk of spinal haematoma: Chaney and Labovsky (2005) describe administering vitamin K to a patient to correct an elevated PT-INR 5 days after cardiac surgery, after which the patient had a thromboembolic stroke. Lim et al. (2006) describe a series of patients who received plasma transfusions to correct elevated PT-INRs: one developed anaphylaxis.

Chung et al. (2011), Fakouri et al. (2009) and Goswami et al. (2011) describe patients who would not be expected to be at a high risk of spinal haematoma, but who developed one anyway. Ladha et al. (2013) present a case of a patient with elevated liver function tests but not coagulopathy before gastrectomy with epidural analgesia. She developed a postoperative deficiency of vitamin K-dependent coagulation factors and required urgent decompression of a spinal haematoma.

Finally, there are two studies describing cases where spinal haematoma was not expected, but was preceded by an abnormal course of events: Ozdemir et al. (2009) describe a patient who developed a late chronic intracranial subdural haematoma after inadvertent dura puncture at epidural catheterization. Walker et al. (2011) describes a spinal haematoma in a patient with normal routine coagulation status but various other risk factors for spinal haematoma: major haemorrhage with massive transfusion, multiple attempts at epidural catheterization including a bloody tap and ROTEM® parameters indicative of coagulopathy.

## Discussion

### General trends of coagulation

This study concerns coagulation after oesophagectomy, which is not as well researched as coagulation after hepatic resection. Nevertheless, our results (Fig. 2) are in concordance with the studies found in the literature review: postoperative coagulation is characterised by moderate increases in PT-INR and aPTT despite these patients being known to be prone to thrombosis. Postoperative inflammation is characterised by thrombocytosis and hyperfibrinogenaemia, which likely compensate for potential deficiencies in coagulation factors that can affect PT-INR and aPTT. Our own research concerning coagulation after major upper gastrointestinal surgery suggest that increases in PT-INR may be caused by postoperative decreases in factor VII while increases in aPTT may be caused by decreases in factor XII. Deficiency of FXII in otherwise healthy individuals is not associated with coagulopathy (Thomas et al. 2016). Given the questionable relevance of standard coagulation parameters, we would agree with other authors, in that an individual assessment of patients’ clinical coagulative states is warranted when routine coagulation parameters are mildly elevated and an epidural catheter needs withdrawing (Gulur et al. 2015).

### The increase in aPTT would not appear to be caused by postoperative renal failure causing accumulation of LMWH

Half of our patients’ postoperative aPTT was above the upper reference limit, which is a greater proportion of patients than that seen in the literature search. The only measure we have of renal function in this study is serum creatinine, which actually decreased over the perioperative period, so it does not explain the prolonged aPTT. A problem with the aPTT assay is that it is not standardised in the same way as PT-INR, so that different methods and reagents can give varying results from a single blood sample (Thomas et al. 2015).

There was an increase in coagulative complications when LMWH was introduced in the USA in a much higher dosage than the one we use (Horlocker and Wedel 1998). It is, however, of note that we give enoxaparin in a standard dose of 40 mg once daily regardless of weight. The patient category most likely to develop spinal haematoma is elderly women undergoing orthopaedic surgery; these patients are much more likely to be small and have renal failure than young patients without significant comorbidity.

### Risk of spinal haematoma

Calculating the risk of an uncommon complication from a set of uncomplicated events is notoriously difficult, as described by Ho et al. (2000) and Hanley and Lippman-Hand (1983). We observed only 130 patients with elevated coagulation results, mainly aPTT, without complication, meaning that we cannot with certainty say that the risk of spinal haematoma was lower than 2.3%; we cannot from the data in this study come to any clinical conclusion regarding whether it is safe to withdraw catheters when the aPTT or PT-INR are mildly elevated.

Is there a place for viscoelastic tests in identifying patients at risk of spinal haematoma on withdrawal of epidural catheters? Two of the studies in the literature review provide cases where ROTEM® or TEG® gave an indication of hypocoagulability when routine tests did not (Simons and Mallett 2007; Walker et al. 2011). On the other hand, ROTEM® is insensitive to anticoagulation with warfarin up to a PT-INR of at least 3.0 (manufacturer’s information) even if viscoelastic tests using specially adapted and, as yet, not commercially available reagents can be used to monitor clinical vitamin K antagonism (Sorensen et al. 2003). Platelet count is recommended in the ASRA guidelines because of the risk of heparin-induced thrombocytopenia after a few days’ treatment with LMWH (Horlocker et al. 2011).

### What is next in this area of research?

The recurring theme of most research in this area since the middle of the 1990s is that the risk of epidural haematoma is small and that routine laboratory tests’ role in risk stratification is unclear. Even the rather large study by Liu et al. (2011), in which epidural catheters were withdrawn from patients with test results that would in most institutions would have been prohibitive, has insufficient power to come to any definitive conclusion about how to interpret abnormal standard test results.

We do not think that more small prospective studies are going to provide us with adequate data allowing a definition of a ‘magic’ values for aPTT or PT-INR which allow withdrawal of epidural catheters without the risk of spinal haematoma. It would be more meaningful to analyse all cases of spinal haematoma such that a definitive identification of risk factors for spinal haematoma could be produced. As already described in the ASRA guidelines, patients at high risk should be clinically monitored much more vigilantly than those at low risk. We propose that patients, who develop spinal haematomas after epidural catheterization or withdrawal of a catheter, should be included in continent-wide studies coordinated by professional bodies, in which blood should be taken and sent for advanced coagulation analysis that is too expensive to be run on patients in prospective studies. It is of great interest to define exactly which, if any, coagulopathy causes spinal haematoma: if non-coagulative factors are more important than blood test results, attention should be paid to these to prevent unnecessary haemorrhagic complications of neuraxial blockade.

## Conclusion

This is a difficult subject to research and deserves considerable respect since epidural catheterization is a procedure that may reduce morbidity and mortality after high-risk operations, but which is rarely immediately life-saving. The data presented in this study support previous research which indicates that major operations are followed by thrombocytosis combined with aPTT and PT-INR suggesting mild coagulopathy. The implications for the risk of spinal haematoma when epidural catheters are removed are still unclear, which is why a prospective and at least continental-wide study investigating patients who have actually had spinal haematoma should be conducted.

## Additional files

Additional file 1: Graphical representation of epidemiological, quality and other laboratory data. Stars indicate significant differences between pre- and postoperative results as tested using Student’s *t* test (*P* < 0.05). aPTT, activated partial prothrombin time. PT-INR, prothrombin time international normalised ratio. Plc, platelet count. EPI, epidural analgesia. IV-PCA, intravenous patient-controlled analgesia with morphine. (TIFF 1498 kb)

Additional file 2: Summaries of the individual articles included in the literature search (Vandermeulen et al. 1994; Sandhu et al. 2000; Greaves 1997; Liu and Mulroy 1998; Horlocker 2003; Moen and Irestedt 2008; Singh et al. 2009; Davies 2007; Shontz et al. 2009; Cwik 2012; Ladha et al. 2013; Okuda and Kitajima 2001; Schulz-Stubner et al. 2005; Tyagi and Bhattacharya 2002; Unic-Stojanovic et al. 2012). (PDF 99 kb)

Additional file 3: Raw data presented as a tabulated text file (.txt). Patients’ ages and the dates of operation have been removed to preserve anonymity. (TXT 91 kb)

## Abbreviations

Alb: Serum albumin
aPTT: Activated partial thromboplastin time
Crea: Serum creatinine
EPI: Epidural analgesia
Hb: Blood haemoglobin
IV-PCA: Intravenous patient-controlled analgesia
LMWH: Low molecular weight heparin
NSAID: Non-steroidal anti-inflammatory drug
Plc: Platelet count
PT-INR: Prothrombin time international normalised ratio
ROTEM®: Rotational thromboelastometry
TEG®: Thromboelastography
