# Supplementary figures and images for: Pre- to postoperative coagulation profile of 307 patients undergoing oesophageal resection with epidural blockade over a 10-year period in a single hospital: implications for the risk of spinal haematoma
Source: Perioper Med (Lond). 2017 Oct 4;6:14. doi: 10.1186/s13741-017-0070-7 (PMC5628458; doi:10.1186/s13741-017-0070-7)

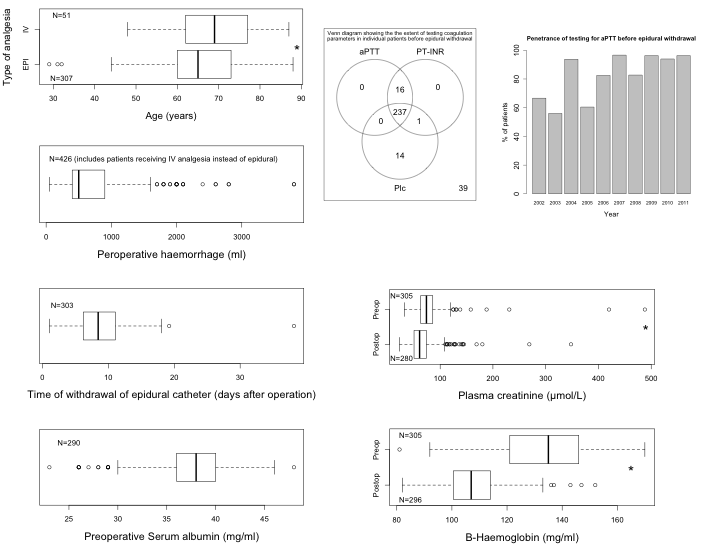

Supplement: Supplementary file 1 — Graphical representation of epidemiological, quality and other laboratory data. Stars indicate significant differences between pre- and postoperative results as tested using Student’s t test (P < 0.05). aPTT, activated partial prothrombin time. PT-INR, prothrombin time international normalised ratio. Plc, platelet count. EPI, epidural analgesia. IV-PCA, intravenous patient-controlled analgesia with morphine. (TIFF 1498 kb) [file 13741_2017_70_MOESM1_ESM.tiff]
