# Supplementary material for: Pre- to postoperative coagulation profile of 307 patients undergoing oesophageal resection with epidural blockade over a 10-year period in a single hospital: implications for the risk of spinal haematoma
Source: Perioper Med (Lond). 2017 Oct 4;6:14. doi: 10.1186/s13741-017-0070-7 (PMC5628458; doi:10.1186/s13741-017-0070-7)
Supplement: Supplementary file 2 — Summaries of the individual articles included in the literature search (Vandermeulen et al. 1994; Sandhu et al. 2000; Greaves 1997; Liu and Mulroy 1998; Horlocker 2003; Moen and Irestedt 2008; Singh et al. 2009; Davies 2007; Shontz et al. 2009; Cwik 2012; Ladha et al. 2013; Okuda and Kitajima 2001; Schulz-Stubner et al. 2005; Tyagi and Bhattacharya 2002; Unic-Stojanovic et al. 2012). (PDF 99 kb) [file 13741_2017_70_MOESM2_ESM.pdf]

Summaries of the articles concerning specific aspects of laboratory tests.			
**Author and year**	**Title**	**Type of article**	**Main conclusions**
Thomas 2015	Thromboelastometry versus free-oscillation rheometry and enoxaparin versus tinzaparin: an in-vitro study comparing two viscoelastic haemostatic tests' dose-responses to two low molecular weight heparins at the time of withdrawing epidural catheters from ten patients after major surgery.	Ex vivo comparison of rotational thromboelastometry and free-oscillation rheometry's responses to increasing doses of timzaparin and enozaparin.	Both thromboelastometric and FOR measures of clot initiation were sensitive to increasing doses of LMWH. Enoxaparin has less effect on clot initiation than tinzaparin when given in equivalent doses of anti-Xa activity: anti-FXa activity does not give a global measure of LMWH's activity. There is significant inter-individual variation in response to LMWH.
Thomas 2013	Rotational thromboelastometry and multiple electrode platelet aggregometry in four patients with abnormal routine coagulation studies before removal of epidural catheters after major surgery: a case series and research study.	Prospective pilot study comparing routine tests of coagulation with ROTEM® and Multiplate®	Four cases of abnormal coagulation found among a series of 20 patients who had undergone major gastrointestinal surgery. Thromboelastometry and tests of platelet count and function indicate normo- to hypercoagulation whereas some routine tests indicate normo- to slight hypocoagulation. ROTEM® could ascribe a prolonged aPTT to heparin effect from a contaminated line.
Nishiyama 2012	Applicability of a compact PT-INR measuring device CoaguChek XS to perioperative management.	Prospective comparison of routine and a novel patient-near method of measuring PT-INR.	There was a good correlation between routine and patient-near PT-INR in ten patients such that this apparatus could be used in the operating theatre instead of sending blood to the hospital laboratory.
Hepner 2002	Coagulation status using thromboelastography in patients receiving warfarin prophylaxis and epidural analgesia.	Prospective observational.	52 patients undergoing elective knee arthroplasty were included. Thromboelastometry (TEG®) was compared to PT-INR at the time of withdrawal of epidural catheters. All patients started taking warfarin the night before surgery. Pre- to postoperatively, PT-INR increased significantly to 1.48. TEG® reaction time increased significantly but was still within the normal range at the time of withdrawal of catheters. No clinical haemorrhage was observed.
Klein 2000	Thromboelastography as a perioperative measure of anticoagulation resulting from low molecular weight heparin: a comparison with anti-Xa concentrations.	Prospective observational study	24 patients undergoing orthopaedic surgery with epidural analgesia and with thrombosis prophylaxis with enoxaparin were included. Routine tests of coagulation, anti-FXa activity, activated clotting time (ACT) and TEG® were consecutively run at several points in time over the perioperative period in order to 'capture' at least one peak and trough of anti-FXa activity following doses of enoxaparin. There was a good correlation between both TEG® reaction- and clot formation times and anti-FXa activity. The authors conclude that TEG® could tentatively be used to measure the degree of anticoagulation provided by LMWH.

Summaries of the articles concerning incidence and risk-factors for haemorrhage.			
**Author and year**	**Title**	**Type of article**	**Main conclusions**
Canto 2002	Thoracic epidurals in heart valve surgery: neurologic risk evaluation.	Prospective observation study of 305 patients.	Same protocol as Pastor 2003 (see below), but prospective and including patients undergoing valve surgery. No clinical spinal haematomas.
Davis 2012	Three-times-daily subcutaneous unfractionated heparin and neuraxial anesthesia: a retrospective review of 928 cases.	Retrospective review	115 (=12%) had an aPTT of >40s at any time before withdrawal of their epidural catheter. In 78, the aPTT decreased after withholding a dose of UFH. The others' aPTT remained between 40-45s, 6 patients' aPTT was above 45s. All were withdrawn without complication. The authors state that there are only 5 published case studies of spinal haematoma caused by withdrawal of epidural catheters during thrombosis prophylaxis with UFH.
Franchi 2011	Coagulation testing before epidural analgesia at delivery: Cost analysis	Retrospective observational study and cost analysis in obstetric epidurals for labour.	7697 patients were included; PT-INR and aPTT were measured in 2871 patients. Fewer than 1% had deranged results and all were given an epidural anyway. Screening with PT-INR and aPTT in this population is therefore unnecessary and very costly. The authors conclude that coagulation tests should only be ordered when there is a clinical indication such as a positive patient or family history of bleeding, or in preeclampsia or haemorrhage.
Gulur 2015	Retrospective analysis of the incidence of epidural haematoma in patients with epidural catheters and abnormal coagulation parameters.	Retrospective review of patient documentation	A period of 7 years covering 11600 epidural catheters (all indications included). 315, ie 3% were removed in the setting of abnormal coagulation, defined as a PT-INR >1.4, aPTT > 35s or Plc<$100x10^6$.. Two epidural haematomas occurred following withdrawal of catheters, which is equivalent to 1:315 (95% confidence interval for true incidence: 1:87-1:2597). Both of these patients had had an epidural and spinal drain for operation of open aortic abdominal aneurysm and had abnormal coagulation parameters at the time of withdrawal of the catheter. The one patient had abnormal PT-INR and aPTT and multiple attempts at spinal catheterization, the other was treated with both LMWH and warfarin.
Horlocker 2003	Thromboprophylaxis and neuraxial anesthesia.	Review concerning risk of spinal haematoma as result of neuraxial blockade.	-
Kassis 2000	The safe use of epidural anesthesia after subcutaneous injection of low-dose heparin in general abdominal surgery.	Prospective cohort study	5000E unfractionated heparin (UFH) was given subcutaneously to fifty patients scheduled for bowel surgery. aPTT, anti-FXa activity and anti-FII activity were measured before, 2 and 4 hours after the injection. Levels of anti-FXa and anti-FIIa activity were not higher than 0.2U/ml in any patient leading to the conclusion that manipulation of epidural catheters ought to be safe 2 hours after UFH injection. aPTT did not increase significantly after the dose of UFH.
Leonard 2000	Evaluation of the effects of levobupivacaine on clotting and fibrinolysis using thromboelastography.	In vitro test using blood from healthy volunteers.	Levobupivacaine decreased TEG® Maximal amplitude more than saline when added to whole blood in a dilution similar to that which might be present at epidural blood patch, but not the systemic concentration provided by a bolus of local anaesthetic given in an epidural catheter.

Summaries of the articles concerning incidence and risk-factors for haemorrhage.

Author and year	Title	Type of article	Main conclusions
Liu 2011	Uncomplicated removal of epidural catheters in 4365 patients with international normalized ratio greater than 1.4 during initiation of warfarin therapy.	Prospective study of patients treated with postoperative warfarin, whose epidurals were withdrawn regardless of PT-INR.	The authors' reasoning is that they suggest that the FVII level is crucial to coagulation, and that there is a lag between PT-INR increasing and FVII decreasing. Epidural catheters were withdrawn when the PT-INR was as high as 7.1. No patient developed a spinal haematoma. The authors conclusion is that the levels of vitamin K dependent coagulation factors are probably sufficient despite high PT-INR. Carvalho replies that this conclusion is not appropriate since the absence of haematomas in the subset with a PT-INR over 3.0 is compatible to 95% certainty, with a risk of haematoma of 3%, which is not an acceptable risk.
Moen 2008	Neurological complications following central neuraxial blockades in obstetrics.	Review article.	Review of complications in obstetric epidurals, not specific to postoperative analgesia.
Pace 2014	Epidurals in patients receiving thromboprophylaxis with unfractionated heparin three times a day: the value of activated partial thromboplastin time testing	Retrospective study of 3500 patients' laboratory results over 2 years. Mixed surgery.	Patients' epidurals were withdrawn around the 3rd postoperative day. Of the 714 patients receiving UFT 5000U 3 times daily, only 20 (=2.7%) had an aPTT >35s and all had risk factors for coagulopathy including pre-existing liver dysfunction or liver resection, administration of SC UFH for ≥5 days and blood product or starch transfusions. The authors conclude that screening with aPTT is unnecessary and costly.
Pastor 2003	Thoracic epidural analgesia in coronary artery bypass graft surgery: seven years' experience.	Retrospective observational study of 714 patients	Patients received an epidural catheter at a level of T1 to T3, 60 minutes before full heparinization for cardiac surgery. Withdrawal of the catheters was performed around day 4 after surgery when routine tests of coagulation were normal. No clinical spinal haematomas occurred. The risk of spinal haematoma is therefore with 95% certainty less than 1/238. The authors suggest that venous haemorrhage is not dangerous because the blood vessel will be compressed by the haematoma before spinal compression occurs. They suggest that blood gas measurement can be used to exclude arterial bleeding in the case of a bloody tap. The authors report that the rate of failure of epidural analgesia was 0, which is not consistent with other literature.
Pumberger 2013	An analysis of the safety of epidural and spinal neuraxial anesthesia in more than 100,000 consecutive major lower extremity joint replacements	Retrospective identification of spinal haematomas in 100,000 patients undergoing lower limb orthopaedic surgery over 10 years.	8 patients had radiologically confirmed haematoma or gas collection, giving a upper 95% confidence interval for risk of haematoma of 1:12,500. The epidural catheters were removed on day 1 or 2 and the diagnosis of haemorrhage or gas collection was on day 3+/-1.5 days. All had either major pain or neurological deficit. PT-INR was 'within the normal range' of up to 2.5. Only 1 patient was 'low risk'. All had taken a drug that affects platelets (5 NSAID's, 1 tricyclic antidepressant, 1 an antiplatelet drug). One had hereditary thrombocytopenia, one had a Plc of $70x10^6$/ml. 2 had excessive alcohol consumption. All had preoperative hypertension. 5 had scoliosis. 6 were treated with postoperative warfarin.
Singh 2009	Factor XI deficiency and obstetrical anesthesia.	Case series of 13 obstetric patients who received an epidural catheter.	FXI deficiency is not an absolute contraindication to neuraxial blockade but should be administered in consultation with a haematologist.
Volk 2012	Incidence of spinal haematoma after epidural puncture: analysis from the German network for safety in regional anaesthesia.	Retrospective registry study including 33,142 non-obstetric epidurals	Incidence of spinal haematoma was 1:6,628. Additionally, one intracranial haematoma occurred. Suspected risk factors were renal function, female sex and type of surgery. In one of the cases, the apparent source of bleeding was 3 levels above the site of puncture, suggesting that the catheter tip may have perforated a blood vessel here.

Summaries of the articles concerning postoperative coagulation.			
**Author and year**	**Title**	**Type of article**	**Main conclusions**
Benzon 2010, Horlocker 2010	Factor VII levels and international normalized ratios in the early phase of warfarin therapy.<br><br>Safe epidural catheter removal in the patient receiving warfarin: does anybody really know what (prothrombin) time it is?	Prospective observational study of 121 patients in whom PT-INR and FVII levels were measured on inter alia post-operative day 1 after joint surgery.	Benzon et al conclude that VII is the most important factor antagonized by warfarin and that there is a lag in its antagonism after initiation of warfarin therapy as thrombosis prophylaxis after surgery. Most of the patients they observed with PT-INR of over 1.4 had adequate levels of FVII (9 out of 11 patients). The authors conclude that withdrawal of epidural catheters on the first postoperative day regardless of PT-INR 'may be safe.'<br><br>Horlocker replies that there were still a significant number of individuals who had inadequate levels of FVII and does not agree with Benzon's conclusion.
Borromeo 2000	Epidural catheter and increased prothrombin time after right lobe hepatectomy for living donor transplantation.	Case series	5 patients who donated a liver segment. Coagulation changes consistent with the other post-hepatectomy studies in this review.
Choi 2005	The changes in coagulation profile and epidural catheter safety for living liver donors: a report on 6 years of our experience.	Retrospective observational study	Almost the same study as Weinberg. 360 patients undergoing hepatic resection for donation were included, 242 of which received an epidural catheter. Serial tests were taken. Plc reached its nadir on day 2-3, PT-INR and aPTT on day 3. No spinal haematomas were observed. During the postoperative course, 1.4% had a Plc of under $80x10^6$/ml and 2.5% had a PT-INR of >2.0.
Davies 2007	Perioperative epidural anaesthesia and analgesia--an appraisal of its role.	Editorial	Discussion of the risks and benefits of epidural analgesia.
Elterman 2015	Coagulation profile changes and safety of epidural analgesia after hepatectomy: a retrospective study	Retrospective study of 141 patients undergoing liver resection.	PT-INR increased significantly to a mean of around 1.5 on postoperative day 2 and then decreased to 1.2 by postoperative day 7. A third of patients received vitamin K or PCC to normalize PT-INR to <1.4 before planned withdrawal of epidural catheters. Although Hb and Plc decreased significantly from pre- to postoperative tests, no patients needed a transfusion of platelets.
Jolly 2011	4 postoperative changes in coagulation parameters in 105 patients undergoing live donor hepatectomies-implications for epidural analgesia.	Retrospective observational study. Conference abstract.	89% of patients deceloped a derangement in PT-INR (>1.4), aPTT (>45s) or Plc ($<100x10^6$/ml) but they had recovered in 83% of cases by the fifth postoperative day.
Karna 2015	Postoperative coagulopathy after live related donor hepatectomy: Incidence, predictors and implications for safety of thoracic epidural catheter	Retrospective study of 100 patients' routine laboratory results after hepatectomy.for organ donation.	Coagulopathy after donor hepatectomy was most common on post-operative day 2 when 84 of the patients had a PT-INR>1.5 and 12 had thrombocytopenia. By day 5 only 14 of the patients were coagulopathic: 10 had an elevated PT-INR and 6 thrombocytopenia. Independent predictors of coagulopathy were low body mass index, small remnant liver and long duration of surgery. 'Coagulopathy' was defined as PT-INR>1.5 or Plc$<100x10^6$/ml.
Matot 2002	Epidural anesthesia and analgesia in liver resection.	Prospective observational study of 136 patients undergoing hepatic resection	Similar dynamics of PT and Plc as the other studies in this review. PT and Plc normalied more quickly in the patients who underwent a minor liver resection compared to those who underwent a major liver resection.
Mohammed 2013	Rotational thromboelastometry and standard coagulation tests for live liver donors.	Prospective comparison of serial ROTEM®, aPTT, PT-INR, Plc and fibrinogen in 50 patients undergoing hepatectomy for donation with epidural analgesia.	ROTEM® disagreed with routine tests and did not show the temporary hypocoagulability suggested by PT-INR and aPTT. None of the tests showed hypercoagulability in this study.

Summaries of the articles concerning postoperative coagulation.

Author and year	Title	Type of article	Main conclusions
Ramspoth 2014	Risk factors for coagulopathy after liver resection	Retrospective cohort study	Coagulopathy followed hepatic resection occurred in only 34% of 146 patients. Of the rest, around 20% (7/146) had a persisting coagulopathy after a week whereas 33/147 experienced a transient coagulopathy. Predictors of postoperative coagulopathy were preoperatively elevated PT-INR, elevated postoperative lactate, and larger resection. Preoperatively elevated liver function tests and peroperative requirement for transfusion of red blood cells or plasma were also associated with postoperative coagulopathy. 'Coagulopathy' was defined as PT-INR>1.3, Plc < $80x10^6$/ml or aPTT>38s.
Schumann 2004	Altered hematologic profiles following donor right hepatectomy and implications for perioperative analgesic management.	Prospective observational study of 8 patients undergoing hepatic resection for donation.	In addition to thoracic epidural analgesia, patients received a peroperative wound infiltration of local anaesthetic. The dynamics of PT, aPTT and Plc were similar to the other studies in this review. No spinal haematoma was observed.
Shontz 2009	predisposing to coagulopathy in patients receiving epidural analgesia for hepatic surgery.	Retrospective chart review of 49 patients who received an epidural for analgesia after hepatic surgery.	Almost half of the patients developed a postoperative coagulopathy as measured by PT-INR (>1.4), aPTT(>40s) or Plc<$100x10^6$/ml. Those with coagulopathy were significantly more likelty to have bled significantly during operation or to have resected a larger volume of liver. Most coagulopathies had corrected by day 5.
Siniscalchi 2004	Increased prothrombin time and platelet counts in living donor right hepatectomy: implications for epidural anesthesia.	Prospective observational study of 30 healthy patients undergoing hepatic resection for donation.	The dynamics of coagulation test results were consistent with the other studies in this review. There was a significant correlation between amount of blood loss, volume of fluids given and extent of liver resection, and PT. No spinal haematoma was observed.
Stamenkovic 2011	Epidural analgesia and liver resection: postoperative coagulation disorders and epidural catheter removal.	Retrospective observational study including 140 consecutive patients who underwent hepatic resection.	Thrombocytopenia troughed on the first postoperative day ($205x10^6$/ml, SD 72); PT-INR and aPTT on the second (1.9 SD 0.5 and 38s SD5.8). Some patients still had abnormal results for these tests on the 7th postoperative day. Epidural catheters were withdrawn when PT-INR was <1.2, On the fifth postoperative day, the average PT-INR was 1.5 +/- 0.36.
Tsui 2004	Delayed epidural catheter removal: the impact of postoperative coagulopathy.	Retrospective observational analysis of 413 patients	Included were patients who underwent various types of surgery. 26 (=6%) had deranged routine coagulation test results such that withdrawal of their catheters was delayed. The dynamics of coagulation test results showed the same pattern as other studies: peak PT-INR and aPTT on day2- 3 and Plc nadir on day 3. Patients who had undergone hepatectomy were significantly more likely to have abnormal postoperative coagulation resulting in delaying of withdrawal of their epidural catheters: a third of these patients had coagulopathy delaying withdrawal of epidural catheters. Among patients undergoing other surgical procedures, between 5% and 15% of patients had abnormal laboratory results leading to delayed withdrawal. No spinal haematoma was observed.
Weinberg 2007<br><br>Bergman 2007	Postoperative changes in prothrombin time following hepatic resection: implications for perioperative analgesia.<br><br>Prothrombin time is not the whole story.	Retrospective observational study of 127 patients who underwent hepatectomy.<br>Reply by Bergman.	PT increased postoperatively, peaking on day 1. Predictive factors for elevated PT were duration of surgery and mass of liver resection. PT remained slightly elevated during the first week. Bergman replies that these patients are generally prone to thrombosis, possibly due to deficiencies in antithrombin or protein C such that 'PT is not the whole story'.
Yuan 2012	Abnormal coagulation profile after hepatic resection: the effect of chronic hepatic disease and implications for epidural analgesia.	Retrospective review of 153 patients who had undergone hepatic resection.	Routine coagulation status consists of PT, aPTT and Plc. The postoperatvive period was divided into an early (day 1-3) and a late (day 1-4) period. The percentage of patients with abnormal routine coagulation status peaked on day 2 (40%) and decreased to 5% on the seventh postoperative day. Risk factors for abnormal coagulation were cirrhosis, preoperative coagulopathy, major resection and intraoperative blood loss.

Summaries of the articles describing case studies or other relevant topics

Author and year	Title	Type of article	Main conclusions
Chaney 2005	Thoracic epidural anesthesia and cardiac surgery: balancing postoperative risks associated with hematoma formation and thromboembolic phenomenon.	Case report	A patient who was given vitamin K to correct an elevated PT-INR before withdrawal of his epidural catheter five days after cardiothoracic surgery. He unfortunately developed a thromboembolic stroke.
Chung 2011	Epidural hematoma occurred by massive bleeding intraoperatively in cesarean section after combined spinal epidural anesthesia -A case report.	Case report.	Case description of an obstetric epidural catheter that was placed under normal conditions for caesarean section. Perioperative massive haemorrhage resulted in coagulopathy and the patient developed a spinal haematoma.
Cwik 2012	Postoperative considerations of neuraxial anesthesia	Review	General review of benefits and risks of epidural anaesthesia.
Fakouri 2009	Spinal epidural hematoma after insertion of a thoracic epidural catheter in the absence of coagulation disorders--a call for raised awareness.	Case report	Case report of a patient who developed a spinal haematoma after epidural catheterization, in the absence of risk factors or traumatic puncture.
Goswami 2011	Epidural haematoma: Rare complication after spinal while intending epidural anaesthesia with long-term follow-up after conservative treatment	Case report	Case of a healthy 25 year old who developed spinal haematoma after epidural analgesia for labour. Conservative treatment and full recovery after 5 years.
Ladha 2013	Spinal haematoma after removal of a thoracic epidural catheter in a patient with coagulopathy resulting from unexpected vitamin K deficiency	Case report	Case description of a patient who underwent gastrectomy due to a tumour. Elevated liver tests but normal coagulation preoperatively. Subjectively uncomplicated surgical course and the epidural was withdrawn on day 3. LMWH restarted 3 hours after withdrawal of catheter. A day later, acute back pain. Diagnosis of spinal haematoma and urgent decompression, before which PT-INR was 2.5 and aPTT 49s. Coagulation analysis found low FVII and FIX. Speculation of whether preoperative malnutrition + antibiotics could be responsible for this.
Lim 2006	Postoperative coagulopathy after liver resection--implications for epidural analgesia.	Audit correspondence.	Five of the 12 patients included received a plasma infusion to correct moderately elevated PT's. One developed anaphylaxis with tachycardia and hypotension.
Okuda 2001	Epidural hematoma in a parturient who developed disseminated intravascular coagulation after epidural anesthesia	Case report	-
Özdemir 2009	Intracranial chronic subdural haematoma as a complication of epidural anesthesia.	Case report	Case of an otherwise healthy 39 year old woman who developed a chronic subdural haematoma after epidural analgesia for scar resection. Catheter placement was complicated by dura puncture. Diagnosis was 21 days after operation.
Shultz-Stubner 2005	Regional analgesia in the critically ill.	Review	Now somewhat outdated review referring principially to guidelines now no longer applicable guidelines.
Simons 2007	Use of thromboelastography to demonstrate persistent anticoagulation after stopping enoxaparin	Case report	A patient with renal failure and previous pulmonary embolus. Routine coagulation tests were within normal limits, anti-FXa was 0.28U/ml but TEG® showed a significant heparin effect. Surgery was delayed and uneventful.
Tyagi 2002	Central neuraxial blocks and anticoagulation: a review of current trends	Review	Compilation of now mainly outdated guidelines.
Unic-Stojanovic 2013	Benefits, Risks and Complications of Perioperative Use of Epidural Anesthesia	Review Article	General review of benefits and risks of epidural anaesthesia.
Walker 2011	Use of thromboelastometry in the assessment of coagulation before epidural insertion after massive transfusion.	Case report	Soldier who received an epidural catheter after massive haemorrhage and transfusion. Placement was complicated by both multiple puncture attempts and blood in the catheter. Routine coagulation tests were normal but ROTEM® EXTEM and INTEM had low MCF before attempts at catheter placement began. FIBTEM-MCF was normal. The patient developed a spinal subdural haematoma, diagnosed on day 10. The authors draw attention to that platelet function may not be normal after massive transfusion and that routine tests of coagulation are not enough to be sure of normal coagulation.
